# A zoom into the nanoscale texture of secondary cell walls

**DOI:** 10.1186/1746-4811-10-1

**Published:** 2014-01-10

**Authors:** Tobias Keplinger, Johannes Konnerth, Véronique Aguié-Béghin, Markus Rüggeberg, Notburga Gierlinger, Ingo Burgert

**Affiliations:** 1 Institute for Building Materials, ETH Zurich, Zurich, Switzerland; 2 Applied Wood Research Laboratory, Empa - Swiss Federal Laboratories for Material Testing and Research, Duebendorf, Switzerland; 3 Department of Materials Science and Process Engineering, BOKU-University of Natural Resources and Life Science, Konrad Lorenz Strasse 24, A-3430, Tulln, Vienna, Austria; 4 INRA-UMR-614 Fractionnement des Agro-Ressources et Environnement (FARE), F-51686, Reims, France; 5 Université de Reims Champagne-Ardenne, UMR614 Fractionnement des Agro-Ressources et Environnement (FARE), F-51686, Reims, France

**Keywords:** Secondary cell walls, Scanning near field optical microscopy, Atomic force microscopy, Diffraction limit, Lignification

## Abstract

**Background:**

Besides classical utilization of wood and paper, lignocellulosic biomass has become increasingly important with regard to biorefinery, biofuel production and novel biomaterials. For these new applications the macromolecular assembly of cell walls is of utmost importance and therefore further insights into the arrangement of the molecules on the nanolevel have to be gained. Cell wall recalcitrance against enzymatic degradation is one of the key issues, since an efficient degradation of lignocellulosic plant material is probably the most crucial step in plant conversion to energy. A limiting factor for in-depth analysis is that high resolution characterization techniques provide structural but hardly chemical information (e.g. Transmission Electron Microscopy (TEM), Atomic Force Microscopy (AFM)), while chemical characterization leads to a disassembly of the cell wall components or does not reach the required nanoscale resolution (Fourier Tranform Infrared Spectroscopy (FT-IR), Raman Spectroscopy).

**Results:**

Here we use for the first time Scanning Near-Field Optical Microscopy (SNOM in reflection mode) on secondary plant cell walls and reveal a segmented circumferential nanostructure. This pattern in the 100 nm range was found in the secondary cell walls of a softwood (spruce), a hardwood (beech) and a grass (bamboo) and is thus concluded to be consistent among various plant species. As the nanostructural pattern is not visible in classical AFM height and phase images it is proven that the contrast is not due to changes in surfaces topography, but due to differences in the molecular structure.

**Conclusions:**

Comparative analysis of model substances of casted cellulose nanocrystals and spin coated lignin indicate, that the SNOM signal is clearly influenced by changes in lignin distribution or composition. Therefore and based on the known interaction of lignin and visible light (e.g. fluorescence and resonance effects), we assume the elucidated nanoscale structure to reflect variations in lignification within the secondary cell wall.

## Background

Structure, chemistry and mechanics of the polymer assembly of secondary cell walls have been studied for decades, because of the high social, environmental and economic relevance of the plant material in classical applications (e.g. wood, paper). More recent research activities are driven by the utilization of lignocellulosic biomass for bioenergy [1-3] and novel biomaterials [4]. The organization of secondary cell walls at the micro-level, with its three-layered and multilamellar structure for wood species and bamboo respectively, is well understood. However we are still lacking knowledge on the organization at the nanoscale in particular about the spatial arrangement and interaction of the different polymers within the cell wall. Most secondary cell walls of xylem cells are made up of the three dominating cell wall polymers cellulose, lignin and hemicelluloses. Cellulose fibrils with a diameter of 3-4 nm are arranged in bigger agglomerates (fibril bundles) with a size of 20-25 nm and are embedded in a matrix consisting of lignin and hemicelluloses [5,6]. During the last decades different cell wall models on the spatial arrangement of the macromolecules within the secondary cell walls have been developed. The micellar theory of Nägeli [7] (developed in the 19^th^ century) refers to a spatial distribution of the macromolecules in a concentric lamellae structure due to alternating circumferential layers of higher cellulose and higher lignin content. This theory has been supported in various studies based on visible/ultraviolet microscopy, the delamination behavior of wood fibers, the distribution and form of pores after selective removal of lignin as well as electron microscopy and atomic force microscopy studies [8-11]. A slightly different model was supposed by Kerr and Goring [12] in terms of concentric arrangement of batches of higher cellulose and lignin content resulting in a segmented lamella structure. Based on high resolution field emission scanning electron microscopy studies on fractured wood samples and cell wall degradation by fungi, an alternative radial agglomeration of cellulose structures was proposed [13,14]. More recently, electron microscopy and atomic force microscopy (AFM) studies supported a random texture without any structured arrangement of the wood components [15,16].

All introduced models are mainly based on electron microscopy studies [11,12,14,17] or on AFM experiments [5,9,10,16]. Both methods are excellent in providing structural information with high resolution, but their potential to provide chemical information is quite limited. Alternative techniques that are strong in chemical analysis of cell walls such as Raman spectroscopy [18] are limited in the required spatial resolution due to the diffraction limit (Rayleigh criterion). To probe structure and chemistry on the nano-level at the same time, scanning probe microscopy has to be combined with spectroscopic techniques [19,20]. One possible combination is Scanning Near-Field Optical Microscopy (SNOM), which has already been developed in the late nineteen-twenties based on the first considerations on breaking the diffraction limit in microscopy [21]. A sample is scanned by a gold or silver coated optical tip, into which a laser is coupled in, with a subwavelength aperture at the end of the optical fiber. The generated signal is not affected by the constraints of diffraction and provides additional photo-optical and thus chemical information. It is based on evanescent waves, which are characterized by rapidly decaying amplitudes very close to the surface (nm range) [22-24]. Although SNOM has been used for the characterization of various biological samples such as proteins, lipids, polysaccharides, DNA and cells [25,26], intact secondary plant cell walls have not been analyzed yet. Therefore the aim was to reveal new insights into the chemical structure of secondary plant cell walls with the help of Scanning Near Field Optical Microscopy by zooming into the nanoscale texture of cell walls of a hardwood, a softwood and a grass.

## Results and discussion

**Figure 1:**
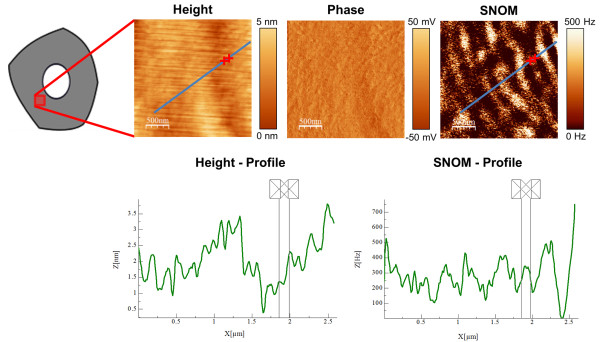
**Height/Phase/SNOM Image of a cross section of beech and the corresponding SNOM and height profiles.** The height and the phase images of the cross section of beech wood (measurement position within the secondary wall (grey) is illustrated) show a very homogeneous surface in contrast to the SNOM image which reveals a clearly segmented circumferential lamellar structure and elongated nodule like features of different sizes. The height and SNOM profile indicate that the SNOM signal does not go along with the height signal (exclusion of height artifacts) and additionally the achieved resolution with SNOM of 120 nm is determined by using the FWHM (Full Width at Half Maximum) of the SNOM signal peak marked in the images and in the profiles.

Figure 1 shows AFM height and phase as well as a SNOM image and the corresponding SNOM/height profile of the marked line of the secondary cell wall layer of a cross section of beech. The height image and the phase image indicate a homogeneous surface (roughness of the height image: 1.1 nm) with only some marginal scratches in the height image which are a result of the microtome cutting. In contrast the SNOM image reveals clearly a segmented circumferential lamellar structure with stripe like features of varying sizes (about 100-200 nm in thickness and 200-700 nm in width).

**Figure 2:**
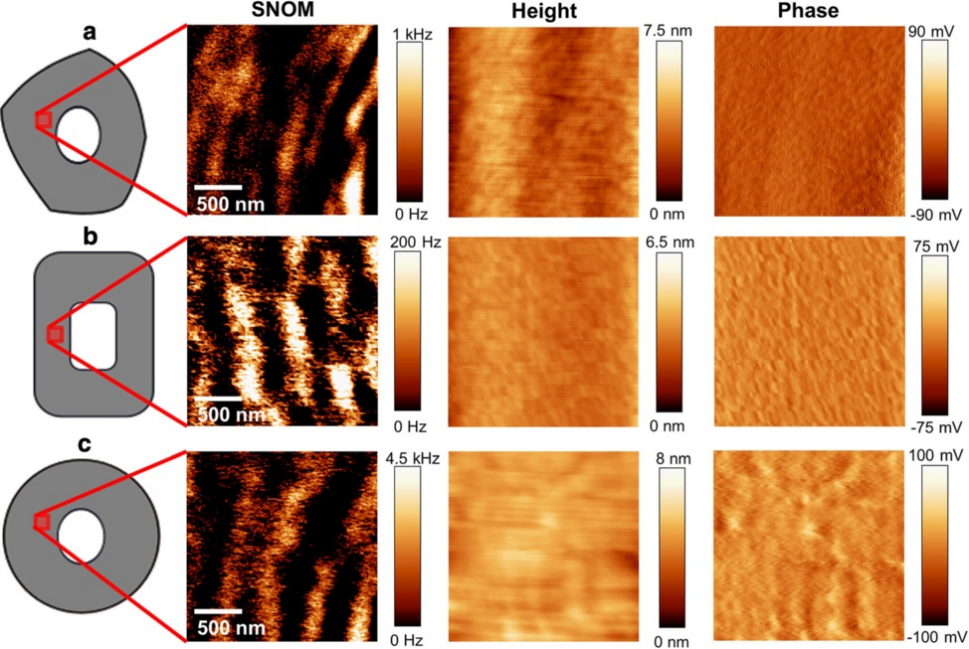
**SNOM/Height/Phase images of beech (a), spruce (b) and bamboo (c).** The SNOM images of the cross sections of the three plant species (positions of the measurements are illustrated) consistently show a circumferential segmentation, in contrast to the homogeneous height and phase images, which points to compositional changes at the nano-level of the secondary cell wall.

**Figure 3:**
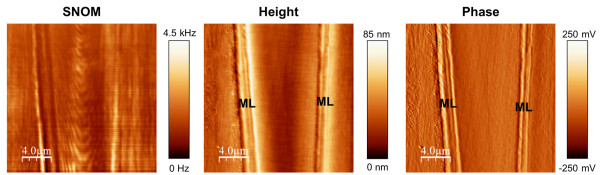
**SNOM/Height/Phase images of a longitudinal section of spruce.** In the height and phase image of the longitudinal section a homogeneous structure apart from the middle lamella (ML) is revealed. The SNOM image of the slightly tilted longitudinal cut shows curl like structures which are in good agreement with the circumferential segmentation in the cross sections.

**Figure 4:**
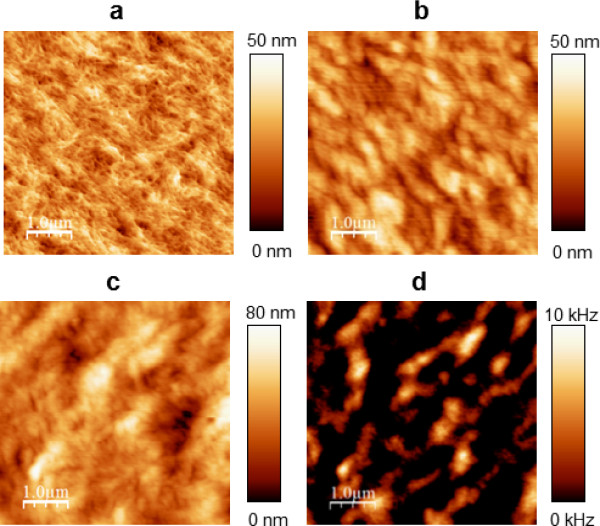
**Height images of the network of casted cellulose nanocrystals measured with an AFM (a)/SNOM (b) tip and Height (c)/SNOM (d) image of the bilayer system (cellulose nanocrystals and lignin).** In the height image of the casted cellulose, measured with the AFM tip, the nanocrystal network is clearly visualized **(a)**; in contrast to the topography of the same sample revealed by the SNOM tip **(b)**. The height image of the bilayer **(c)** reveals the typically formed globular structures of lignin together with the underlying network of the nanocrystals. In contrast in the SNOM image of the bilayer **(d)** only the globules of the lignin macromolecules and their agglomerations can be seen.

SNOM images of cross sections of beech (Figure 2a), spruce (2b) and bamboo (2c) cell walls indicate that the circumferential segmentation in light and dark batches is a common feature for different dicot and monocot species, despite differences in cell organization and chemical composition. The cell wall of both wood species possesses a segmented concentric lamellar pattern with a size of around 125 nm in thickness and 200 to 750 nm in width. In bamboo the same pattern is visible but with a slightly larger width (500-800 nm) and thickness of the segments (ca. 150 nm). Another remarkable feature is that the elucidated structures appear differently curved in dependence of cell form and positioning of the SNOM scanning (compare beech at different positions in Figure 1 and Figure 2a). This is a strong indication for the superimposed circumferential pattern of the cellulose/lignin architecture in the cell walls, which is also supported by the fact that the structures can be seen in other anatomical directions. In Figure 3 the SNOM, height and phase images of a longitudinal cut of spruce wood are shown. The SNOM image reveals a curl like structure of the secondary cell wall due to the slightly tilted cutting direction. This structure is not visible in the height and phase image proofing that the contrast is based on differences in the chemical composition/structure. To gain a deeper understanding of the SNOM signal origin in secondary cell wall analysis, we conducted a comparative investigation on a bilayer of cellulose nanocrystals and spin coated DHP (DeHydrogenation-Polymer)- lignin on top. Figure 4a shows the AFM-height image of the network of cellulose nano-crystals after casting on a quartz substrate–measured with a standard tuning fork AFM tip and Figure 4b the same sample measured with a SNOM tip. It is obvious that the spatial resolution with the SNOM tip is smaller compared to the resolution with the AFM tip–nevertheless the overall roughness remains identical (same z-range of the scan). The reduced resolution can be explained as the tip diameter of the SNOM is a couple of times larger (≈100 nm) and in addition the Q-factor is lower.

Figure 4c shows the topography of the self-assembled lignin model compounds on top of the cellulose nanocrystal film. The images indicate that the lignin forms globular structures (bigger z-range compared to Figure 4b) in the size of 250-500 nm on top of the cellulose nanocrystals which is in good agreement with the results of previous publications [27-29]. Remarkably, the underlying structure of the cellulose nanocrystals is still visible. In contrast, the SNOM image of the bilayer (Figure 4d) reveals only the individual globules of lignin macromolecules and their agglomerations.

**Figure 5:**
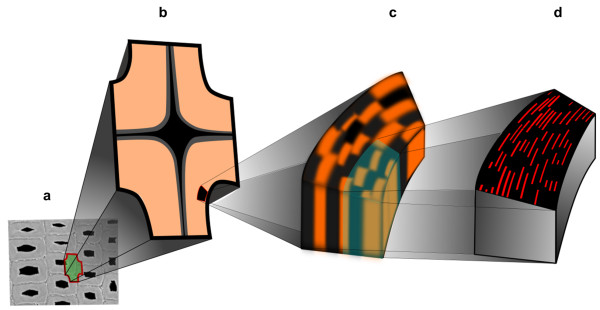
**Model of the macromolecular assembly based on the SNOM measurements.** In Figure 5**a** latewood cells of spruce are shown and in 5**b** an illustrated zoom in the layered structure of the secondary cell wall. 5**c** reflects the measured segmented lamellar structure in the S2 layer and 5**d** illustrates the presumably underlying structure (red: cellulose fibrils; black: matrix consisting of lignin and hemicelluloses) according to the cell wall model introduced by Kerr and Goring [12].

In general SNOM offers the possibility to use all the contrast mechanisms of conventional confocal microscopy e.g.: birefringence, elastic scattering, Raman effect and fluorescence [30]. In view of the specific chemical composition of the cell wall components it is most likely that lignin contributes more strongly to the SNOM signal than cellulose and hemicelluloses, due to its interaction with light (autofluorescence and resonance effects) [31-33]. Hence, based on the observations on the model substances it seems reasonable to assume that the SNOM signal predominantly visualizes changes in the lignin amount or composition with high sensitivity. Therefore the nanostructural pattern observed within the cell walls of beech, spruce and bamboo is supposed to be due to variations in lignification, either in amount or chemical structure. The achieved resolution of around 100 nm (limited to the size of the aperture), most probably does not reflect the exact size of the regions of lignin variations. A smaller segmented lamellar structure with structures in the 10 nm range as introduced in the model by Kerr and Goring [12] would presumably result in a similar pattern, since the sizes of the batches cannot be precisely determined (Figure 5).

## Conclusions

The SNOM images provide for the first time sub-diffraction limited chemical information on the spatial distribution of the cell wall components in secondary cell walls. The circumferential pattern of cellulose and lignin rich regions is likely to be the result of spatial constraints and progression of the cell wall formation process. The superimposed circumferential structure is due to the cellulose fibril spinning process by cellulose synthase complexes [34], which lay down the cellulose scaffold in a circumferential manner. The subsequent lignification in a radical polymerization process is likely to locally produce segments of higher and lower degrees of lignin or alterations in the chemical structure [35-37], which become evident in the SNOM images. A striking observation is the incidence of the distribution pattern among different plant species, including a representative of hardwoods, softwoods and grasses. This points to a rather universal principle of spatial cellulose and lignin assembly in secondary cell walls, and is therefore highly relevant for the understanding of cell wall structure and its enzymatic degradation for energy conversion from lignocellulosic raw materials in general.

## Methods

Air dried specimens of spruce, beech (provided by Oliver Vay, Kompetenzzentrum Holz GmbH, Linz, Austria) and bamboo (from an experimental forest in Miaoshanwu Nature Reserve, Zhejiang Province, China) with dimensions of ~10×10×10 mm^3^ (radial/tangential/longitudinal) were glued onto AFM specimen discs (orientation of the fibrils was taken into account) and afterwards on cylinders due to fixation reasons. A highly smooth surface (1.5 × 1.5 mm^2^) was cut using a ultramicrotome (Reichert Jung–Ultracut) with a diamond knife (DiATOME) and the following cutting parameters: speed: 1 mm/s, clearance angle: 8° and cutting angle: 45°. Sections of initially 2 μm reduced gradually to approximately 50 nm were removed in order to minimize mechanical impact of cutting forces on the surface. Both, wood (beech and spruce) and bamboo samples were not embedded, to avoid any potential influence by the embedding material.

Cellulose nano-crystals (CNCs) were prepared from ramie (*Boehmeria nivea*) fibers and synthetic lignins (Dehydrogenation Polymers - DHP) were synthetisized by peroxidase/hydrogen peroxide mediated polymerization of coniferyl alcohol (guaiacyl G unit), as previously described [27]. 200 μg of CNC was applied to a clean quartz slide (3.14 cm^2^) from CNC aqueous suspension (0.1%, w/v) and dried under vacuum for 12 h. DHP solution (2 gL^-1^) in dioxan/water (9/1, v/v) was then deposited by spin-coating on CNC film with a spin-coater (Speedline Technologies, USA). A volume of 50 μL was deposited on a stationary solid slide, which was then accelerated at 1260 rpm/s and spun at 3000 rpm for 40 s. Thickness and refractive index of CNC film before DHP deposit were respectively 270 nm ± 5 nm and 1.550, measured by spectroscopic ellipsometry (Uvisel, Horiba Jobin Yvon, Palaiseau, France).

**Figure 6:**
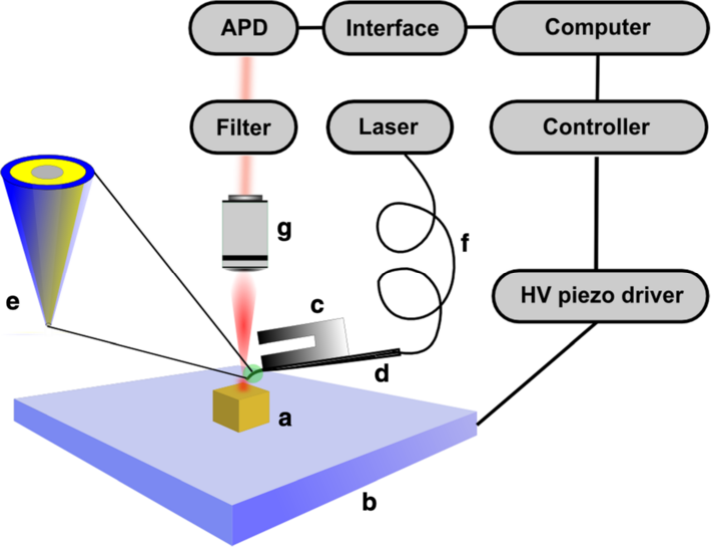
**Scheme of the SNOM-setup showing the arrangement of the main components.** The sample **(a)** is placed on a piezo-scanning stage **(b)** and scanned with a tuning fork SNOM probe **(c)**. Through the SNOM tip **(d)** (zoom **(e)** showing the inner Au/Cr coating) the laser light coupled out of an optical fibre **(f)** is coming in contact with the sample and reflected back through an objective **(g)** and filter before the signal is detected with an APD.

SNOM measurements were performed with a MultiView 2000™ instrument (Nanonics Imaging, Jerusalem, Israel) in reflection configuration in dry state (scheme of the setup in Figure 6). Images shown are a selection out of ≈ 100 SNOM measurements. The tuning fork AFM was operated in phase-feedback and for coupling a gold coated glass tip with an aperture between 50-150 nm (typical resonant frequency of the tip around 35 kHz and a Q factor of around 500) to the 532 nm laser an optical fiber was used. For the AFM measurement in Figure 4a a tuning fork AFM probe with a tip diameter of 20 nm, a resonant frequency of 33.18 kHz and a Q-factor of ≈ 1520 was used. As a detector an Avalanche Photo diode is needed/used (see Figure 6), as the SNOM signal is extremely small, which makes the signal detection highly challenging.

Size of the generated AFM/SNOM images varies between 20 × 20 μm^2^- 2.5 × 2.5 μm^2^ at a resolution of 256 × 256 pixels and 12 sub-steps between each pixel. Further information on the spatial resolution of the SNOM and AFM mode of the instrument can be found on http://www.nanonics.co.il. For processing the acquired data were exported from the measurement software (Quartz, Faraday Instruments) to WSxM 4.0 develop 11.6 software. All images were parabola flattened, equalized and the contrast was adjusted for visibility reasons [38].

## Abbreviations

AFM: Atomic Force Microscopy; CNC: Cellulose Nano-Crystals; DHP: Dehydrogenation Polymers; FT-IR: Fourier Transform Infra-Red Spectroscopy; SNOM: Scanning Near-Field Optical Microscopy; TEM: Transmission Electron Microscopy.

## Competing interests

The authors declare that they have no competing interests.

## Authors’ contributions

TK, JK, NG and MR performed experiments and analyzed data. VAB provided samples. TK, NG and IB co-wrote the paper. All authors discussed results and commented on the manuscript. All authors read and approved the final manuscript.
